# Facile synthesis of novel graphene sponge for high performance capacitive deionization

**DOI:** 10.1038/srep08458

**Published:** 2015-02-13

**Authors:** Xingtao Xu, Likun Pan, Yong Liu, Ting Lu, Zhuo Sun, Daniel H. C. Chua

**Affiliations:** 1 Engineering Research Center for Nanophotonics & Advanced Instrument, Ministry of Education, Department of Physics, East China Normal University, Shanghai 200062, China; 2 Department of Materials Science and Engineering, National University of Singapore, Singapore 117574

## Abstract

Capacitive deionization (CDI) is an effective desalination technique offering an appropriate route to obtain clean water. In order to obtain excellent CDI performance, a rationally designed structure of electrode materials has been an urgent need for CDI application. In this work, a novel graphene sponge (GS) was proposed as CDI electrode for the first time. The GS was fabricated via directly freeze-drying graphene oxide solution followed by annealing in nitrogen atmosphere. The morphology, structure and electrochemical performance of GS were characterized by scanning electron microscopy, Raman spectroscopy, nitrogen adsorption-desorption, X-ray photoelectron spectroscopy, cyclic voltammetry and electrochemical impedance spectroscopy. The electrosorption performance of GS in NaCl solution was studied and compared with pristine graphene (PG). The results show that due to the unique 3D interconnected porous structure, large accessible surface area and low charge transfer resistance, GS electrode exhibits an ultrahigh electrosorption capacity of 14.9 mg g^−1^ when the initial NaCl concentration is ~500 mg L^−1^, which is about 3.2 times of that of PG (4.64 mg g^−1^), and to our knowledge, it should be the highest value reported for graphene electrodes in similar experimental conditions by now. These results indicate that GS should be a promising candidate for CDI electrode.

Capacitive deionization (CDI), also known as electrosorption, has attracted enormous attention in recent years as an energy saving and environmentally friendly desalination technique, because it can be conducted at ambient conditions and low voltages (<2 V) without secondary waste, and doesn't require high-pressure pumps, membranes, distillation columns, or thermal heaters^1^,^2^,^3^,^4^,^5^,^6^,^7^,^8^,^9^. As an electrochemical water treatment method, CDI is developed based on the principle of electric double-layer (EDL) capacitor. With an external electrostatic field supply between electrodes, the charged ions can move toward oppositely charged electrodes, and be attracted within the EDL formed between the solution and the electrode interface (Figure 1a). Therefore, the ion adsorption capacity of an electrode is directly related to the physical properties and internal structure of the electrode materials, such as electrical conductivity, surface areas and pore size^10^,^11^.

Generally, CDI electrodes are typically made of porous carbon materials, such as activated carbon (AC)^12^,^13^,^14^, carbon nanotubes (CNTs)^15^,^16^,^17^,^18^,^19^, carbon aerogel (CA)^20^,^21^,^22^, carbon fibers (CNFs)^23^,^24^,^25^, mesoporous carbon (MC)^26^,^27^ and others. Among these carbon species, graphene with a flexible planar structure (ultrathin layer), high specific surface area (theoretically ~2600 m^2^ g^−1^) and superior electron mobility (theoretically ~2.5 × 10^5^ cm^2^ V^−1^ s^−1^ at room temperature), has been theoretically and experimentally demonstrated to possess superior CDI performance^28^,^29^,^30^,^31^,^32^,^33^,^34^. However, due to the strong agglomeration between graphene nanosheets during the reduction process, pristine graphene (PG) had relatively low specific surface area, and thus showed low electrosorption capacity of 0.45–1.85 mg g^−1^ 28,29,30, which hampers its application in CDI. To circumvent this problem, different methods have been adopted to prevent the agglomeration of graphene sheets in order to enhance their CDI performance. For example, by introducing some guest materials, such as pyridine^31^, CNTs^35^,^36^, MC^26^, AC^37^ and resol^38^,^39^ as “spacers” between graphene sheets to form a sandwich structure, the electrosorption capacity (0.83–3.23 mg g^−1^) was enhanced obviously compared with PG. Nevertheless, it was difficult to keep the “spacers” dispersed uniformly between the graphene sheets, so the aggregation of graphene still partly existed in these composites^40^. Therefore, further efforts are still needed to design and optimize the structure of graphene for its practical application in CDI. Recently, a three-dimensional (3D) macroporous graphene architecture with wide pore size distribution was fabricated by using polystyrene microspheres as sacrificial templates, and it possessed large surface area, high pore volume and thus achieved high electrosorption capacity of 3.9 mg g^−1^ 32. Another work carried out by *Yan et al.*^1^ reported a sponge-templated strategy to prepare graphene with high surface area and favorable size distribution, and its electrosorption capacity reached a very high value of 4.95 mg g^−1^. There is no denying the fact that the 3D macroporous graphene and sponge-templated graphene can prevent the agglomeration of graphene sheets, and enlarge the specific surface area, and thus improve the electrosorption capacity. However, the synthetic strategies of these materials are relatively complicated, time-consuming, and practically high-cost. Moreover, further enhancement of electrosorption capacity is still necessary in order to meet the demand of practical application of CDI. Hence, seeking a facile and low-cost strategy to fabricate graphene structure with high electrosorption capacity should be an urgent need for current CDI research.

In this work, a novel graphene sponge (GS) was fabricated via directly freeze-drying graphene oxide (GO) solution followed by annealing in nitrogen atmosphere. The obtained GS shows a porous structure with a high specific surface area of 356.0 m^2^ g^−1^. When used as CDI electrode, GS exhibits an ultrahigh electrosorption capacity of 14.9 mg g^−1^ in NaCl solution with an initial concentration of ~500 mg L^−1^.

## Results and discussion

**Figure 1:**
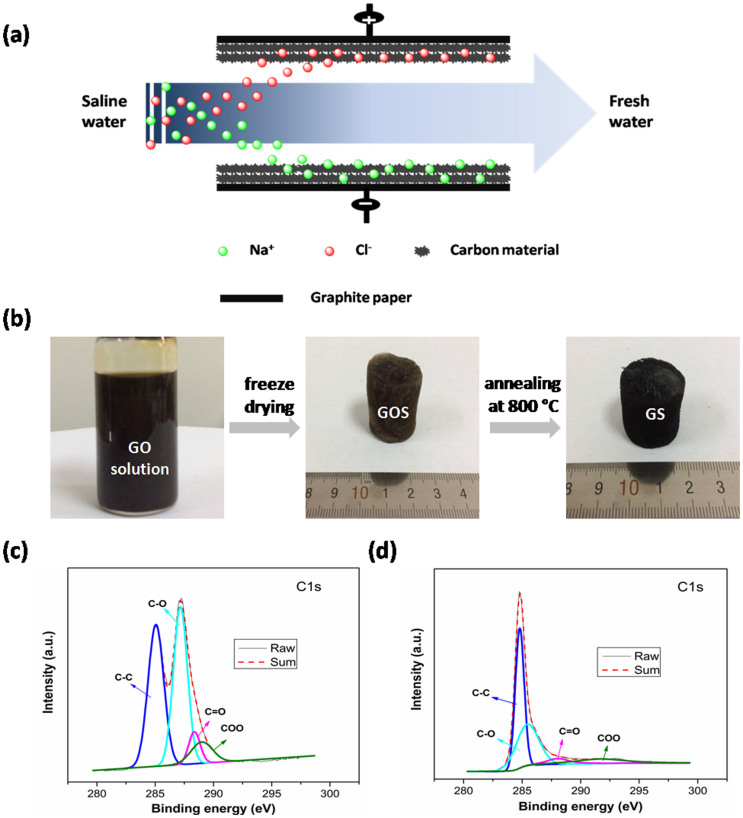
(a) Schematic diagram of the CDI process. The basic principle of CDI involves the application of a voltage (<2.0 V) between two oppositely placed electrodes. Saline water flows through a spacer sandwiched between the oppositely placed electrodes. On the application of an electric potential to the CDI cell, the Na^+^ and Cl^−^ are attracted towards the oppositely charged porous carbon electrodes and subsequently stored inside them. The resulting freshwater thus contains a reduced amount of salt. This phenomenon is similar to energy storage in supercapacitors and batteries. (b) Schematic illustration of the procedure for the preparation of GS; high-resolution C1s XPS of (c) GOS and (d) GS.

Figure 1b illustrates the method for the fabrication of GS. GO sponge (GOS) was produced by freeze-drying process in which the strong interaction of GO in water played an important role. In this process, the interaction of GO was strong enough so that GO solution could be directly frozen to produce the sponge. After annealing, a spongy graphene architecture GS was obtained. Compared with the sponge-templated method reported in the literature^1^, the strategy in this work is more simple, low-cost and easy for the large-scale production. The surface compositions of the GOS and GS were tested by X-ray photoelectron spectroscopy (XPS). From the C 1 s spectrum of the GOS in Figure 1c, the peaks are observed at 285.0, 287.1, 288.4, and 289.0 eV, corresponding to the sp^2^ aromatic rings, and the oxygen-related functional groups C–OH, C = O and COOH, respectively^41^,^42^,^43^. However, these oxygen-related functional groups are sharply weakened in GS, as shown in Figure 1d, indicating most of these groups were effectively removed after annealing. The result is similar to previous report^43^. The XPS analysis confirms that GOS has been successfully reduced to be GS during the annealing process.

**Figure 2:**
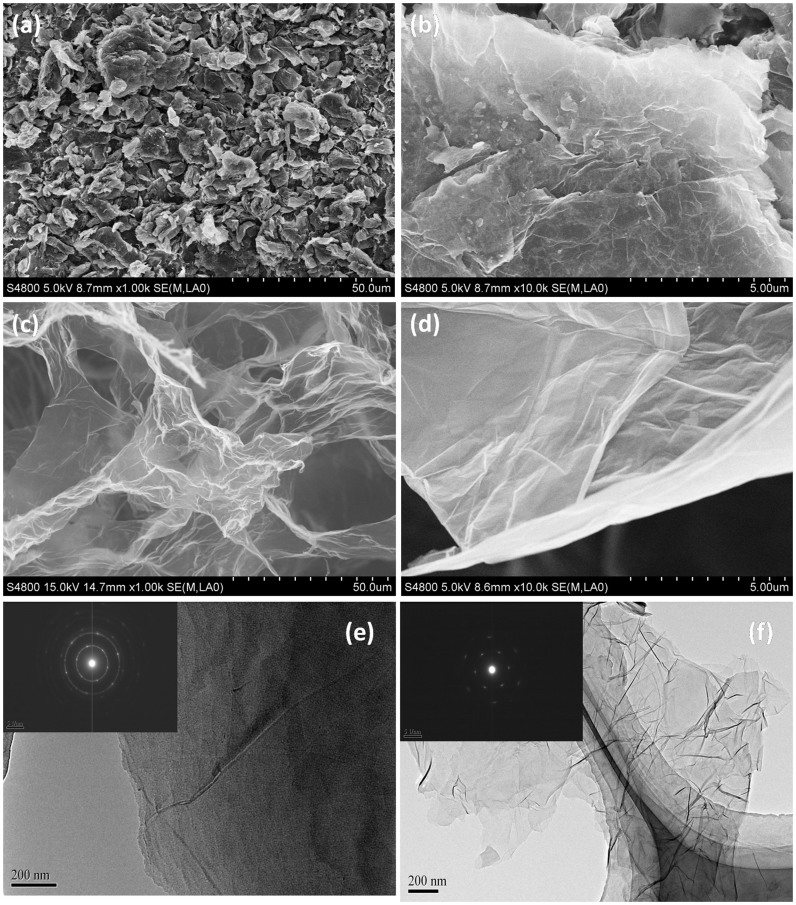
Morphology and structure of GS and PG. (a–d) SEM images of PG (a, b) and GS (c, d) at low and high magnification. (e, f) TEM images of PG (e) and GS (f). Insets of (e) and (f) are SAED patterns of PG and GS, respectively.

Figure 2a, b show the morphology of PG at low and high magnification, respectively. As shown, the PG shows a plane structure with some graphene sheets stacking to graphite platelets due to the strong Van der Waals forces among individual graphene nanosheets. Nevertheless, as shown in Figure 2c, GS exhibits an interconnected, porous 3D framework of randomly oriented, crinkly sheets. The high magnification scanning electron microscopy (SEM) image in Figure 2d reveals clearly that the obtained 3D GS structure is comprised with corrugated and scrolled graphene nanosheets. These curls and wrinkles clearly act to prevent graphene sheets from restacking together with each other. Furthermore, the microstructures of GS and PG were investigated by transmission electron microscope (TEM), as shown in Figure 2e, f. It can be observed that PG shows a multilayer graphene structure with some graphene sheets stacking to graphite platelets (Figure 2e). The selected area electron diffraction (SAED) result indicates that PG has a polycrystal structure, and the inner circle is brighter than outer circle (inset of Figure 2e), demonstrating that PG is composed of multilayer graphene sheets^44^,^45^. While exfoliated graphene sheets of GS with random sizes can be identified by folds and wrinkles in the structures (Figure 2f), and in fact, the crumpled surface and edge of graphene sheets are beneficial to ion adsorption and make full use of the surface area of graphene^46^. The SAED pattern of GS shows a hexgonal dffraction dots (inset of Figure 2f), indicating that GS has few layered structure^47^,^48^,^49^. It is known that the EDL on the large exposed area can provide sufficient accessible sites for ions accumulation and the space between the pores can serve as an efficient channel for fast mass transfer^1^. Therefore, the GS is expected to be a desirable electrode material for high performance CDI.

**Figure 3:**
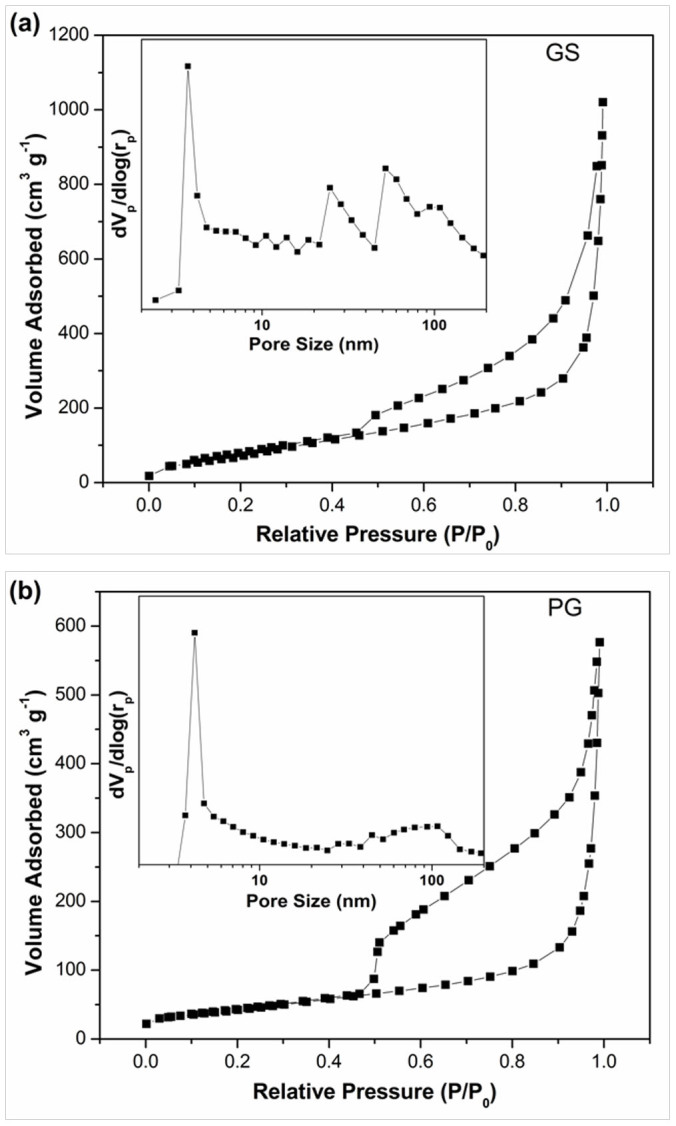
Nitrogen sorption isotherms of (a) GS and (b) PG. Insets are the pore size distribution of GS and PG, respectively.

The pore structure and specific surface area of the samples were tested by nitrogen adsorption–desorption isotherms, as shown in Figure 3. Both GS and PG show a typical type IV hysteresis loop as defined by IUPAC, which is characteristic of mesoporous materials^11^. The hysteresis loop which appears at lower relative pressure (0.4–0.8) indicates the presence of mesopores and at higher relative pressure (0.8–1.0) is attributed to macropores^32^. As calculated, GS displays a high specific surface area of 356.0 m^2^ g^−1^ with a pore volume of 1.51 cm^3^ g^−1^, which is about twice of those of PG (150.5 m^2^ g^−1^ and 0.83 cm^3^ g^−1^). The novel 3D architecture of GS can efficiently prevent the stacking between graphene interlayers, and thus contributes to much higher specific surface area and pore volume, which ensure more sites for ion adsorption during the desalination process. The pore size distribution profiles of GS and PG derived from the desorption branches of the isotherms by using the Barrett–Joyner–Halenda model are shown in the insets of Figure 3a and b, respectively. Compared to PG, GS shows much more mesopores and macropores, which is consistent with the SEM observations. As known, the interconnected macropores within graphene frameworks are favorable for buffering ions to shorten the diffusion distances from the external electrolyte to the interior surfaces^32^,^50^, and the mesopores in thin walls can enhance the ion transport and electrosorption capacity^10^,^50^.

To further confirm that the novel 3D architecture of GS helps to prevent the stacking of graphene sheets, Raman spectroscopy was used to evaluate the thickness and graphitic quality of GS and PG, shown in Supplementary Figure 1. The D-band of graphitic materials is a measure of disorder and arises due to the breathing mode of k-point phonons of A_1g_ symmetry while the G-band is associated with the conjugated structure of sp^2^ carbon domains. The intensity ratio (I_D_/I_G_) of D band and G band for GS is about 1.04, whereas the value for PG is only 0.90, implying a decrease in the average size of the sp^2^ domains and a partially ordered crystal structure in GS compared to that in PG^42^,^51^. Previous works have reported that a higher I_D_/I_G_ is beneficial to the charge transfer in the adsorption process^15^,^52^. Thus, GS is expected to accelerate the charge transfer. Moreover, the 2D band could be used to identify the thickness of graphene sheets^53^,^54^. A broader and asymmetric 2D band usually indicates an increased graphene layers^1^. Clearly, a sharp 2D band is observed for GS compared to PG, suggesting a decreased stacking of graphene sheets, which is in accordance with the SEM and TEM observations. Hence, the Raman results demonstrate that GS has a fewer layered and higher disordered graphitic structure than PG, implying sufficient exfoliation of graphene sheets by using freeze-drying method. Such a structure has been previously reported to help improving the electrical conductivity^1^,^32^.

**Figure 4:**
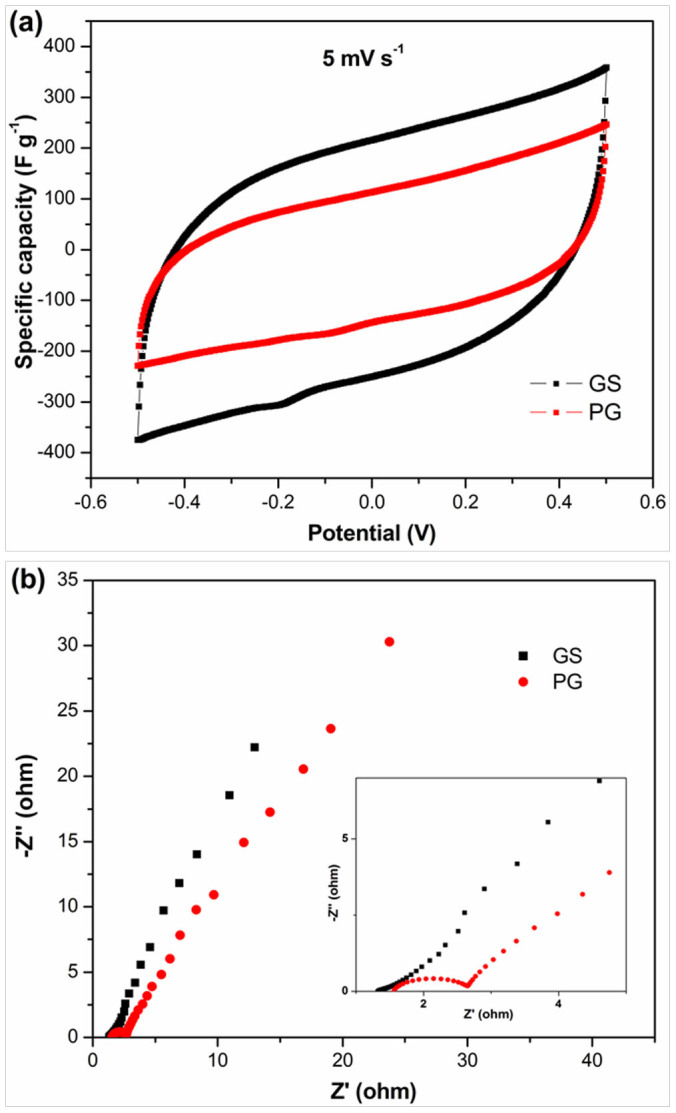
(a) CV curves of GS and PG measured at a scan rate of 5 mV s^−1^ and (b) Nyquist plots of GS and PG electrodes in 1 M NaCl aqueous solution. Inset of (b) is the corresponding expanded high-frequency region of the plots.

Cyclic voltammetry (CV) measurements have been utilized to examine the electrochemical behavior of the electrodes^55^,^56^. Figure 4a shows the CV curve of GS and PG at a scan rate of 5 mV s^−1^ in 1 M NaCl solution with a potential range from −0.5 to 0.5 V. As clearly seen, no obvious Faradaic reaction is observed from the CV curves, which suggests that the CV behavior results from EDL due to the Coulombic interactions, rather than the electrochemical reduction/oxidation reactions^32^. During the annealing process, most of the oxygen-containing groups are removed, so no Faradaic activity is detected during the CV measurements. The specific capacitance of GS electrode calculated from the CV curve is 205.20 F g^−1^, while the one of PG is only 117.31 F g^−1^. The higher specific capacitance of GS electrode can be ascribed to its higher specific surface area and pore volume, fewer layered and higher disordered graphitic structure as well as the excellent conductivity. Therefore, it should be believed that GS shows great superiority for CDI application.

Electrochemical impedance spectroscopy (EIS) analysis has been recognized as one of the principal methods to examine the electrical conductivity of a carbon electrode. The Nyquist profiles of GS and PG electrodes in 1 M NaCl aqueous solution are presented in Figure 4b. It can be obviously seen that the plots display similar shapes, consisting of a linear trait at the low frequency region and a small quasi-semicircle at the high frequency one. The small quasi-semicircle at the high-frequency region is derived from the double layer capacitance (C_dl_) in parallel with the charge transfer resistance (R_ct_)^35^. The R_ct_ can be obtained from the diameter of the semicircle. The R_ct_ for GS is around 0.27 Ω which is much lower than that of PG (1.12 Ω), indicating that GS should have a lower charge-transfer resistance and a superb conductivity.

**Figure 5:**
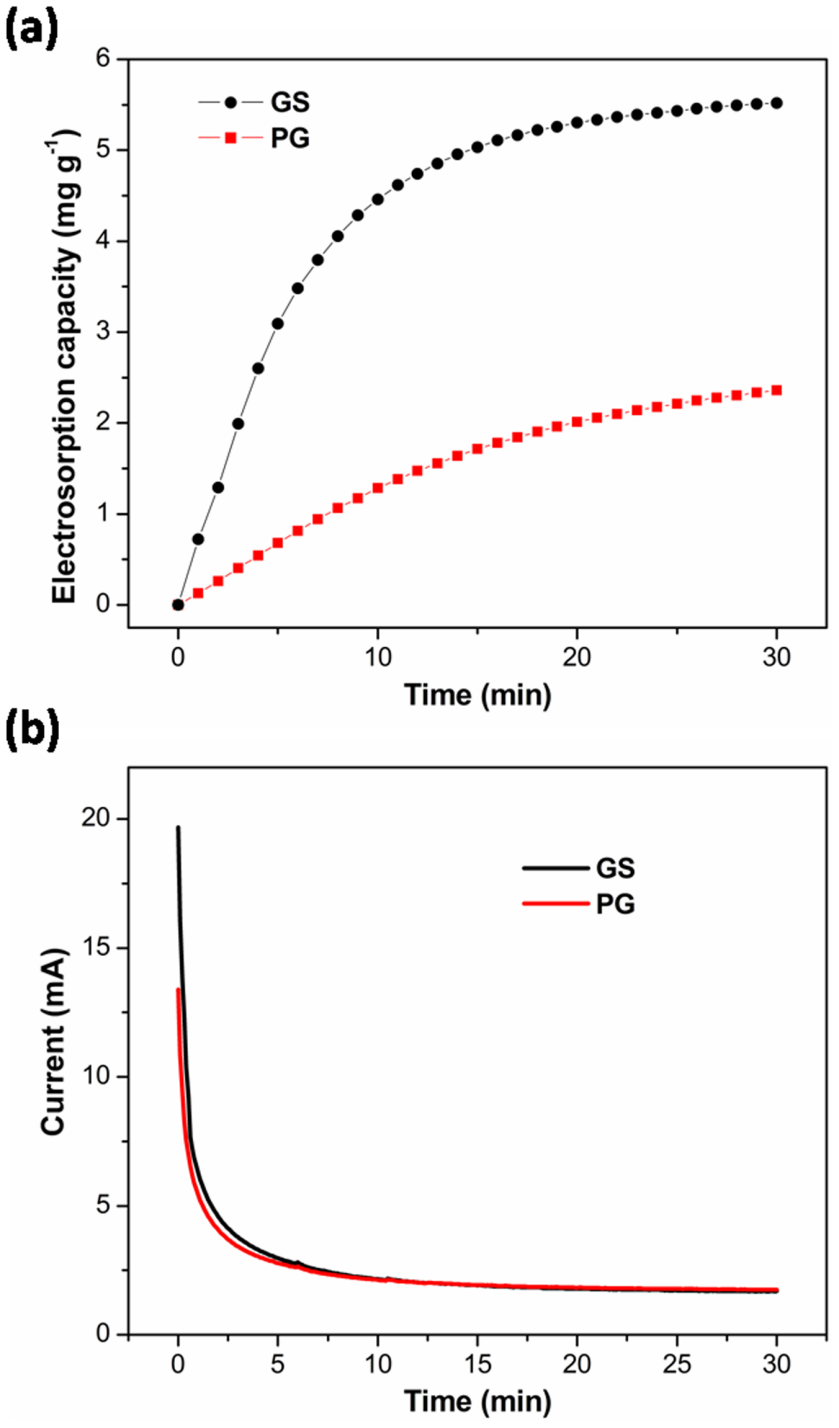
(a) Electrosorption capacity and (b) current transient for GS and PG electrodes over 30 minutes in NaCl solution with an initial concentration of ~50 mg L^−1^ at an applied voltage of 1.5 V.

To determine the electrosorption performance of GS and PG electrodes, batch mode CDI experiments were carried out in NaCl solution with an initial concentration of ~50 mg L^−1^ at an applied potential of 1.5 V. The current variations were recorded simultaneously and independently at each experiment. Figure 5a, b show the electrosorption performances and typical current responses of GS and PG, respectively. Once the electric field was applied, the adsorption amount increased sharply. Then, the change became gradually lower until equilibrium was reached. It can be noted that GS shows an electrosorption capacity of 5.52 mg g^−1^, which is nearly twice of that of PG (2.36 mg g^−1^). To our knowledge, it is highest value reported for graphene structures as CDI electrodes by now (Supplementary Table 1). This is mainly due to the following reasons: (i) during the fabrication process, the freeze-drying method can help to prevent graphene sheets from restacking together with each other and form an interconnected, porous 3D network which is composed of randomly oriented, crinkly sheets. Such a 3D network structure can provide a better contact at the interface between the electrode material and the solution as well as facilitate the charge transfer. (ii) It is well known that surface area is a very important factor for the eletrosorption performance. When compared with graphene structures in the literatures, GS shows the highest value of surface area (Supplementary Table 1) and this high surface area ensures more sites for ion adsorption during the desalination process. (iii) GS shows a suitable pore structure with much more mesopores and macropores. As well known, the interconnected macropores within graphene frameworks are favorable for buffering ions to shorten the diffusion distances from the external electrolyte to the interior surfaces^32^,^50^, and the mesopores in thin walls can enhance the ion transport and electrosorption capacity^10^,^50^.

Charge efficiency (*Λ*) is a functional tool to gain insight into the double layer formed at the interface between the electrode and solution^57^,^58^,^59^,^60^,^61^, as calculated according to the following equation:
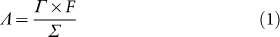
where *F* is the Faraday constant (96485 C mol^−1^), *Γ* is the electrosorption capacity (mol g^−1^) and *Σ* (charge, C g^−1^) is obtained by integrating the corresponding current. According to equation 1, the charge efficiency of GS is determined to be 0.50, which is nearly twice of that of PG (0.27). This should be also due to the 3D porous structure of GS which facilitates the ion diffusion and charge transfer. It should be noticed that the charge efficiencies of both GS and PG electrodes are less than 1, which is ascribed to the following reasons: (i) during the electrosorption process, the co-ions are simultaneously expelled from the electrical double layer accommodating the adsorbed ions, which has a negative effect on the charge efficiency^37^; (ii) the binder used in the electrode fabrication can decrease the effective surface area and conductivity of the electrode^35^. Fortunately, an effective method has been proposed to solve this problem by introducing charge barrier membrane into CDI^62^,^63^,^64^,^65^.

**Figure 6:**
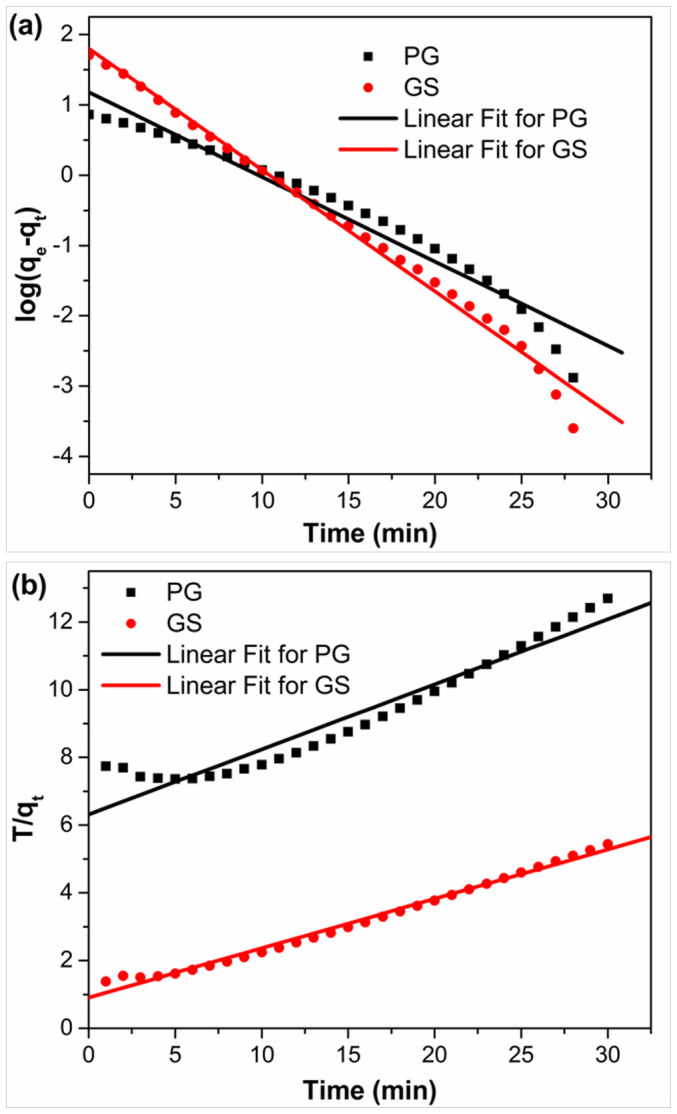
Linear fitting of the electrosorption of NaCl by GS and PG electrodes using (a) pseudo-first-order kinetic equation and (b) pseudo-second-order kinetic equation.

Adsorption kinetics, indicating the adsorption rate, is an important characteristic of adsorbents. It can be determined by using the pseudo-first-order adsorption kinetics and pseudo-second-order adsorption kinetics equations, which are often presented as:
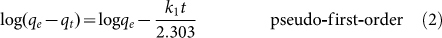

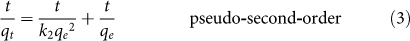
where q_e_ (mg g^−1^) and q_t_ (mg g^−1^) are the amounts of NaCl adsorbed at equilibrium and time t (min), respectively. k_1_ (mg g^−1^ min^−1^) and k_2_ (g mg^−1^ min^−1^) are the adsorption rate constants of pseudo-first-order and pseudo second order equations, respectively. Figure 6 show the linear fitting between the equations and experimental data. The rate constants (as presented in Supplementary Table 2) can be obtained from the slopes and intercepts of the fitting lines in Figure 6. Normally closeness of regression coefficient to 1 supports the assumption of kinetics for the adsorption process. It is found that both of pseudo-first-order and pseudo-second-order kinetics models fit the experimental data for PG, but pseudo-second-order kinetics model describes the electrosorption behavior of GS better. The rate constants for GS and PG using pseudo-second-order kinetics equation are 0.036 and 0.028, respectively. The higher rate constant for GS is ascribed to the quick access of ions onto the surface of GS through the more suitable pore structure^66^.

**Figure 7:**
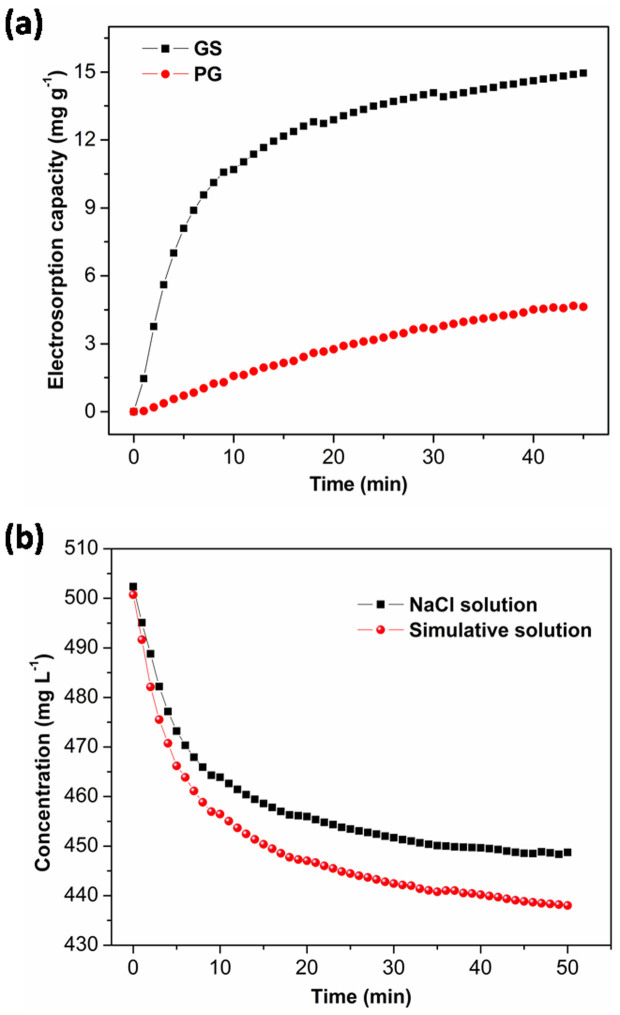
(a) Electrosorption capacities of GS and PG electrodes in NaCl solution; (b) electrosorption performance of GS electrode investigated in NaCl solution and simulative solution. Initial concentration: ~500 mg L^−1^; applied voltage: 1.2 V.

In the actual case, if the Total Dissolved Solids (TDS, measured in mg L^−1^) is higher than 500 mg L^−1^, water is not suitable for drinking. The electrosorption experiments were further performed in NaCl solution with an initial concentration of ~500 mg L^−1^ at an applied voltage of 1.2 V (Figure 7a). The electrosorption capacity of GS is calculated to be 14.9 mg g^−1^, which is about 3.2 times of that of PG (4.64 mg g^−1^). Therefore, GS exhibits better advantages over PG especially on the high salinity deionization. The electrosorption capacity of GS is also compared to those of other carbon electrode material reported in the literatures (Supplementary Table 3). Obviously, though GS has a relatively low specific surface area, it exhibits the highest electrosorption capacity among these carbon electrodes in similar experimental conditions. It is known that actual saline water, especially seawater, contains various ions. Therefore, the electrosorption performance of GS electrode was further tested in a simulative solution with a concentration of 500 mg L^−1^ which has the similar main salt composition with seawater (weight ratio in all salts: NaCl 75.9%, KCl 2.0%, MgCl_2_ 6.4%, CaCl_2_ 6.0% and MgSO_4_ 9.7%). The result was compared with that obtained in NaCl solution (Figure 7b). As shown in Figure 7b, the electrosorption performance of GS electrode in simulative solution is a little better than that in NaCl solution. It has been reported that during the electrosorption process, ions with a higher valence can be more effectively adsorbed because of stronger electrostatic force^29^,^38^. Therefore, due to the presence of divalent ions (Mg^2+^, Ca^2+^ and SO_4_^2−^), GS shows a higher electrosorption performance in simulative solution than that in NaCl solution. This result indicates that GS should be a promising candidate as electrode material for practical CDI process.

**Figure 8:**
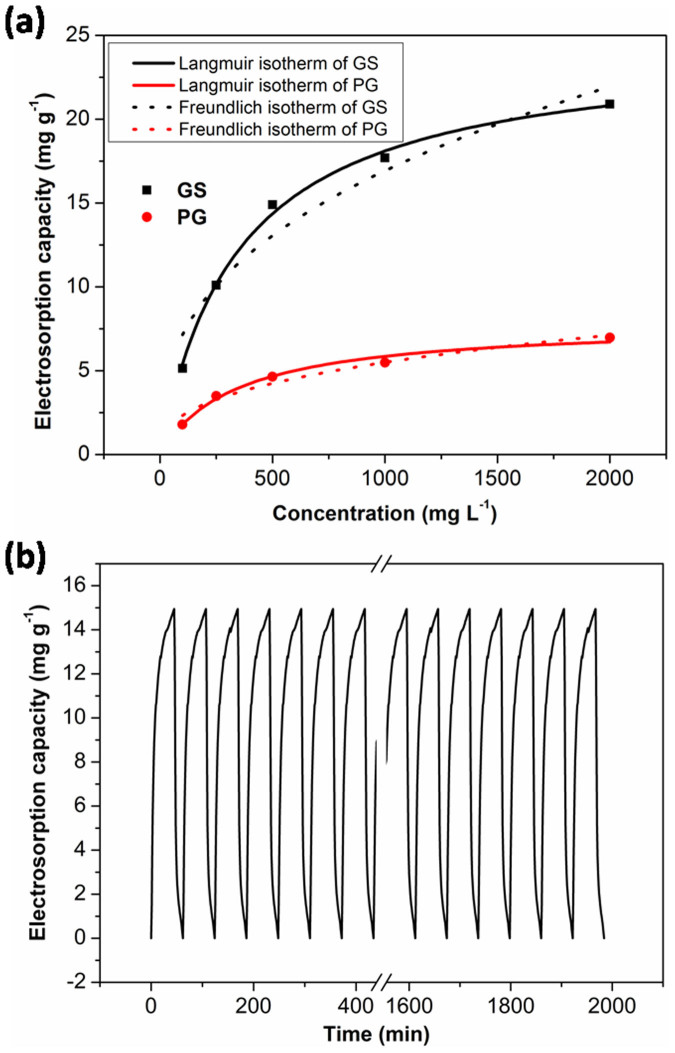
(a) Experimental and fitting data by employing Langmuir and Freundlich isotherms for GS and PG; (b) electrosorption and regeneration cycles of GS in ~500 mg L^−1^ NaCl solution at an applied voltage of 1.2 V.

Further experiments were carried out in NaCl solutions with different initial concentrations to study the electrosorption isotherms of the electrodes. The initial concentrations of NaCl solutions ranged from 100 mg L^−1^ to 2000 mg L^−1^. Fig. 8a shows the electrosorption isotherms of GS and PG. It can be observed that the electrosorption capacity increases as the NaCl concentration is raised, which is due to the enhanced mass transfer rate of ions inside the pores and reduced overlapping effect by higher concentration of solution. Langmuir isotherm (4) and Freundlich isotherm (5) are used to fit the experimental data,
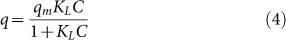

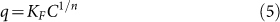
where C is the equilibrium concentration (mg L^−1^), q is the amount of adsorbed NaCl (mg g^−1^), and q_m_ is the maximum adsorption capacity corresponding to complete monolayer coverage (mg g^−1^). 1/n is unitless Freundlich exponent. Supplementary Table 4 shows the determined parameters and regression coefficients r^2^, K_L_ and K_F_ of Langmuir and Freundlich isotherms. It is found that based on higher regression constant r^2^, Langmuir isotherm can describe the electrosorption performance of all electrodes better than Freundlich isotherm.

As known, good regeneration is essential for electrode materials. Therefore, electroadsorption-desorption behavior of GS has also been investigated by repeating several charging (1.2 V) and discharging processes (0 V) in ~500 mg L^−1^ NaCl solution, as shown in Figure 8b. It can be seen that GS electrode can be completely regenerated and reused for over 30 cycles without any declination. This result further confirms that GS is promising to be used as electrode material for actual application of CDI.

## Conclusion

In this work, GS was prepared via directly freeze-drying GO followed by annealing in nitrogen atmosphere, and applied as CDI electrode for the first time. The results show that (i) GS exhibits an interconnected, 3D porous framework with a high specific surface area of 356.0 m^2^ g^−1^ and pore volume of 1.51 cm^3^ g^−1^, much higher than those of PG; (ii) GS shows a ultrahigh electrosorption capacity of 14.9 mg g^−1^ when the initial NaCl concentration is ~500 mg L^−1^, which is about 3.2 times of that of PG (4.64 mg g^−1^) and also higher than those of carbon electrodes reported in the literatures by now; (iii) the enhanced electrosorption performance of GS is ascribed to its large accessible surface area and pore volume, low charge transfer resistance and superior pore structure; (iv) GS should be a promising candidate as electrode material for CDI.

## Methods

### Preparation of GO

GO was prepared according to the method reported in our previous work^28^. In brief, graphite powder was put into a solution of concentrated nitric acid and sulphuric acid (1:2 in volume) and kept at 80°C for 5 h. The mixture was cooled to room temperature, diluted with deionized (DI) water and left overnight. Then, the reaction vessel was immersed in an ice bath, and potassium permanganate was added slowly. Successively, the mixture was stirred and left for 2 h. Then, after the dilution with DI water, 30% H_2_O_2_ was added into the mixture, and the color of mixture changed into brilliant yellow along with bubbling. Finally, the mixture was filtered and washed with HCl aqueous solution (1:10 in volume), DI and ethanol, respectively. Finally, the obtained GO was dried in vacuum oven at 60°C for 24 h.

### Preparation of GS

In a typical process, GO solution (~4 mg mL^−1^) in a vial was frozen by placing it in a freezer at a freezing temperature of −18°C for 2 days. After the GO solution was completely frozen, the vial was moved to a freeze-dryer and dried at a sublimating temperature of −53°C and a pressure <10 pa for 3 days to get the GOS. Finally, GS was obtained by annealing GOS in a tubular furnace at 800°C under nitrogen flow for 3 h. For comparison, PG was fabricated by placing GO in a tubular furnace at 800°C under nitrogen flow for 3 h.

### Characterization

The surface morphology and structure of the samples were characterized by SEM (JEOL JSM-LV5610) and TEM (CM200). The pore size distribution and Brunauer–Emmett–Teller specific surface area were deduced from the nitrogen physical adsorption measurement data which were obtained using (ASAP 2010) Accelerated Surface Area and Porosimetry System (Micrometitics, Norcross, GA). Raman spectra were obtained by Renishaw inVia microscope. A He-Ne laser (633 nm) was used as the light source for excitation. XPS measurement was performed on an Imaging Photoelectron Spectrometer (Axis Ultra, Kratos Analytical Ltd.) with a monochromatic Al Ka X-ray source. CV and EIS measurements were carried out in 1 M NaCl solution by using Autolab PGSTAT 302N electrochemical workstation in a three-electrode mode, including a standard calomel electrode as reference electrode and a platinum foil as counter electrode. The specific capacitance (*C*, F g^−1^) can be obtained from CV curves using the following equation: 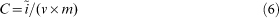
where 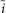 is the average current (A), *v* is the scan rate (V s^−1^) and *m* is the mass of electrodes (g).

### Electrosorption experiments

The electrodes were prepared by mixing 80 wt% of samples, 10 wt% of acetylene black, and 10 wt% of plyvinyl alcohol slurry. The mixtures were pressed onto graphite papers and dried in vacuum oven at 60°C overnight.

The CDI experiments were investigated by batch-mode electrosorption experiments with a continuously recycling system, as described in our previous works^67^. In each experiment, the analytical pure NaCl solution with a volume of 50 mL was employed as the target solution and the flow rate was 27 mL min^−1^. A direct voltage of 1.5 V was applied on the opposite electrodes. The initial concentration of NaCl solution was ~50 mg L^−1^, and the solution temperature was kept at 298 K. The relationship between conductivity and concentration was obtained according to a calibration table made prior to the experiment^28^. The concentration variation was continuously monitored and measured at the outlet of the unit cell by using an ion conductivity meter.

In our experiment, the electrosorption capacity (*Γ*, mg g^−1^) was defined as follows:
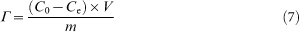
where *C*_0_ and *C*_e_ are initial and final NaCl concentrations (mg L^−1^), *V* is the volume of NaCl solution (L) and *m* is the total mass of the electrodes (g).

## Author Contributions

L.P. and X.X. designed the research, analyzed the data and wrote the manuscript. X.X. and Y.L. performed the characterization. T.L., Z.S. and D.C. discussed the results and commented on the manuscript. All authors reviewed the manuscript.

## Supplementary Material

Supplementary Information Supplementary Information includes Figure and Tables

## References

[b1] Yang Z. Y. *et al.* Sponge-templated preparation of high surface area graphene with ultrahigh capacitive deionization performance. Adv. Funct. Mater. 24, 3917–3925 (2014).

[b2] Yin H. *et al.* Three-dimensional graphene/metal oxide nanoparticle hybrids for high-performance capacitive deionization of saline water. Adv. Mater. 25, 6270–6276 (2013). 23963808 10.1002/adma.201302223

[b3] Wen X., Zhang D., Yan T., Zhang J. & Shi L. Three-dimensional graphene-based hierarchically porous carbon composites prepared by a dual-template strategy for capacitive deionization. J. Mater. Chem. A 1, 12334–12344 (2013).

[b4] Garcia-Quismondo E. *et al.* New testing procedures of a capacitive deionization reactor. Phys. Chem. Chem. Phys. 15, 7648–7656 (2013). 23591701 10.1039/c3cp50514f

[b5] Hojati-Talemi P., Zou L., Fabretto M. & Short R. D. Using oxygen plasma treatment to improve the performance of electrodes for capacitive water deionization. Electrochim. Acta 106, 494–499 (2013).

[b6] Gao X., Omosebi A., Landon J. & Liu K. Enhancement of charge efficiency for a capacitive deionization cell using carbon xerogel with modified potential of zero charge. Electrochem. Commun. 39, 22–25 (2014).

[b7] Demirer O. N., Naylor R. M., Rios Perez C. A., Wilkes E. & Hidrovo C. Energetic performance optimization of a capacitive deionization system operating with transient cycles and brackish water. Desalination 314, 130–138 (2013).

[b8] Oren Y. Capacitive deionization for desalination and water treatment—past, present and future. Desalination 228, 10–29 (2008).

[b9] Sui Z., Meng Q., Zhang X., Ma R. & Cao B. Green synthesis of carbon nanotube–graphene hybrid aerogels and their use as versatile agents for water purification. J. Mater. Chem. 22, 8767–8771 (2012).

[b10] Han L., Karthikeyan K., Anderson M. A. & Gregory K. B. Exploring the impact of pore size distribution on the performance of carbon electrodes for capacitive deionization. J. Colloid Interface Sci. 430, 93–99 (2014). 24998059 10.1016/j.jcis.2014.05.015

[b11] Wang H. *et al.* Design of graphene-coated hollow mesoporous carbon spheres as high performance electrodes for capacitive deionization. J. Mater. Chem. A 2, 4739–4750 (2014).

[b12] Yan C. J., Zou L. D. & Short R. Polyaniline-modified activated carbon electrodes for capacitive deionisation. Desalination 333, 101–106 (2014).

[b13] Nadakatti S., Tendulkar M. & Kadam M. Use of mesoporous conductive carbon black to enhance performance of activated carbon electrodes in capacitive deionization technology. Desalination 268, 182–188 (2011).

[b14] Chen Z., Song C., Sun X., Guo H. & Zhu G. Kinetic and isotherm studies on the electrosorption of NaCl from aqueous solutions by activated carbon electrodes. Desalination 267, 239–243 (2011).

[b15] Nie C. Y. *et al.* Electrophoretic deposition of carbon nanotubes-polyacrylic acid composite film electrode for capacitive deionization. Electrochim. Acta 66, 106–109 (2012).

[b16] Liu Y. *et al.* Enhanced desalination efficiency in modified membrane capacitive deionization by introducing ion-exchange polymers in carbon nanotubes electrodes. Electrochim. Acta 130, 619–624 (2014).

[b17] Nie C. *et al.* Electrophoretic deposition of carbon nanotubes film electrodes for capacitive deionization. J. Electroanal. Chem. 666, 85–88 (2012).

[b18] Dai K., Shi L., Fang J., Zhang D. & Yu B. NaCl adsorption in multi-walled carbon nanotubes. Mater. Lett. 59, 1989–1992 (2005).

[b19] Wang S. *et al.* Equilibrium and kinetic studies on the removal of NaCl from aqueous solutions by electrosorption on carbon nanotube electrodes. Sep. Purif. Technol. 58, 12–16 (2007).

[b20] Jung H.-H., Hwang S.-W., Hyun S.-H., Lee K.-H. & Kim G.-T. Capacitive deionization characteristics of nanostructured carbon aerogel electrodes synthesized via ambient drying. Desalination 216, 377–385 (2007).

[b21] Xu P., Drewes J. E., Heil D. & Wang G. Treatment of brackish produced water using carbon aerogel-based capacitive deionization technology. Water Res. 42, 2605–2617 (2008). 18258278 10.1016/j.watres.2008.01.011

[b22] Suss M. E. *et al.* Capacitive desalination with flow-through electrodes. Energy Environ. Sci. 5, 9511–9519 (2012).

[b23] Wang G. *et al.* Hierarchical activated carbon nanofiber webs with tuned structure fabricated by electrospinning for capacitive deionization. J. Mater. Chem. 22, 21819–21823 (2012).

[b24] El-Deen A. G., Barakat N. A. M., Khalil K. A. & Kim H. Y. Development of multi-channel carbon nanofibers as effective electrosorptive electrodes for a capacitive deionization process. J. Mater. Chem. A 1, 11001–11010 (2013).

[b25] Wang G. *et al.* Activated carbon nanofiber webs made by electrospinning for capacitive deionization. Electrochim. Acta 69, 65–70 (2012).

[b26] Zhang D., Wen X., Shi L., Yan T. & Zhang J. Enhanced capacitive deionization of graphene/mesoporous carbon composites. Nanoscale 4, 5440–5446 (2012). 22836788 10.1039/c2nr31154b

[b27] Tsouris C. *et al.* Mesoporous carbon for capacitive deionization of saline water. Environ. Sci. Technol. 45, 10243–10249 (2011). 22032802 10.1021/es201551e

[b28] Li H. B., Lu T., Pan L. K., Zhang Y. P. & Sun Z. Electrosorption behavior of graphene in NaCl solutions. J. Mater. Chem. 19, 6773–6779 (2009).

[b29] Li H., Zou L., Pan L. & Sun Z. Using graphene nano-flakes as electrodes to remove ferric ions by capacitive deionization. Sep. Purif. Technol. 75, 8–14 (2010).

[b30] Li H., Zou L., Pan L. & Sun Z. Novel graphene-like electrodes for capacitive deionization. Environ. Sci. Technol. 44, 8692–8697 (2010). 20964326 10.1021/es101888j

[b31] Wang H. *et al.* Graphene prepared via a novel pyridine–thermal strategy for capacitive deionization. J. Mater. Chem. 22, 23745–23748 (2012).

[b32] Wang H. *et al.* Three-dimensional macroporous graphene architectures as high performance electrodes for capacitive deionization. J. Mater. Chem. A 1, 11778–11789 (2013).

[b33] Jia B. & Zou L. Graphene nanosheets reduced by a multi-step process as high-performance electrode material for capacitive deionisation. Carbon 50, 2315–2321 (2012).

[b34] Jia B. & Zou L. Wettability and its influence on graphene nansoheets as electrode material for capacitive deionization. Chem. Phys. Lett. 548, 23–28 (2012).

[b35] Li H., Liang S., Li J. & He L. The capacitive deionization behaviour of a carbon nanotube and reduced graphene oxide composite. J. Mater. Chem. A 1, 6335–6341 (2013).

[b36] Zhang D. *et al.* Enhanced capacitive deionization performance of graphene/carbon nanotube composites. J. Mater. Chem. 22, 14696–14704 (2012).

[b37] Li H. B., Pan L. K., Nie C. Y., Liu Y. & Sun Z. Reduced graphene oxide and activated carbon composites for capacitive deionization. J. Mater. Chem. 22, 15556–15561 (2012).

[b38] Wang Z., Yue L., Liu Z.-T., Liu Z.-H. & Hao Z. Functional graphene nanocomposite as an electrode for the capacitive removal of FeCl_3_ from water. J. Mater. Chem. 22, 14101–14107 (2012).

[b39] Wang Z. *et al.* Effective desalination by capacitive deionization with functional graphene nanocomposite as novel electrode material. Desalination 299, 96–102 (2012).

[b40] Chen Y. *et al.* High-performance supercapacitors based on a graphene–activated carbon composite prepared by chemical activation. RSC Adv. 2, 7747–7753 (2012).

[b41] Yang D. *et al.* Chemical analysis of graphene oxide films after heat and chemical treatments by X-ray photoelectron and Micro-Raman spectroscopy. Carbon 47, 145–152 (2009).

[b42] Stankovich S. *et al.* Synthesis of graphene-based nanosheets via chemical reduction of exfoliated graphite oxide. Carbon 45, 1558–1565 (2007).

[b43] Akhavan O. The effect of heat treatment on formation of graphene thin films from graphene oxide nanosheets. Carbon 48, 509–519 (2010).

[b44] Moon I. K., Lee J., Ruoff R. S. & Lee H. Reduced graphene oxide by chemical graphitization. Nature Commun. 1, 73 (2010). 20865806 10.1038/ncomms1067

[b45] Wu X. *et al.* High-rate capacitive performance of graphene aerogel with a superhigh C/O molar ratio. J. Mater. Chem. 22, 23186–23193 (2012).

[b46] Xiong Z., Liao C. & Wang X. G. Self-assembled macroporous coagulation graphene network with high specific capacitance for supercapacitor applications. J. Mater. Chem. A 2, 19141–19144 (2014).

[b47] Robertson A. W. & Warner J. H. Hexagonal single crystal domains of few-layer graphene on copper foils. Nano Lett. 11, 1182–1189 (2011). 21322599 10.1021/nl104142k

[b48] Meyer J. *et al.* On the roughness of single-and bi-layer graphene membranes. Solid State Commun. 143, 101–109 (2007).

[b49] Ding X., Ding G., Xie X., Huang F. & Jiang M. Direct growth of few layer graphene on hexagonal boron nitride by chemical vapor deposition. Carbon 49, 2522–2525 (2011).

[b50] Wu Z.-S. *et al.* Three-dimensional graphene-based macro-and mesoporous frameworks for high-performance electrochemical capacitive energy storage. J. Am. Chem. Soc. 134, 19532–19535 (2012). 23148416 10.1021/ja308676h

[b51] Zhu C., Guo S., Fang Y. & Dong S. Reducing sugar: new functional molecules for the green synthesis of graphene nanosheets. ACS Nano 4, 2429–2437 (2010). 20359169 10.1021/nn1002387

[b52] Pan L. *et al.* Electrosorption of anions with carbon nanotube and nanofibre composite film electrodes. Desalination 244, 139–143 (2009).

[b53] Ferrari A. *et al.* Raman spectrum of graphene and graphene layers. Phys. Rev. Lett. 97, 187401 (2006). 17155573 10.1103/PhysRevLett.97.187401

[b54] Graf D. *et al.* Spatially resolved Raman spectroscopy of single-and few-layer graphene. Nano Lett. 7, 238–242 (2007). 17297984 10.1021/nl061702a

[b55] Liu Y. *et al.* Carbon aerogels electrode with reduced graphene oxide additive for capacitive deionization with enhanced performance. Inorg. Chem. Front. 1, 249–255 (2014).

[b56] Zafra M. *et al.* A novel method for metal oxide deposition on carbon aerogels with potential application in capacitive deionization of saline water. Electrochim. Acta 135, 208–216 (2014).

[b57] Porada S., Zhao R., Van Der Wal A., Presser V. & Biesheuvel P. M. Review on the science and technology of water desalination by capacitive deionization. Prog. Mater. Sci. 58, 1388–1442 (2013).

[b58] Porada S. *et al.* Direct prediction of the desalination performance of porous carbon electrodes for capacitive deionization. Energy Environ. Sci. 6, 3700–3712 (2013).

[b59] Dlugolecki P. & van der Wal A. Energy recovery in membrane capacitive deionization. Environ. Sci. Technol. 47, 4904–4910 (2013). 23477563 10.1021/es3053202

[b60] Zhao R., Biesheuvel P. & Van der Wal A. Energy consumption and constant current operation in membrane capacitive deionization. Energy Environ. Sci. 5, 9520–9527 (2012).

[b61] Zhao R., Biesheuvel P., Miedema H., Bruning H. & Van der Wal A. Charge efficiency: a functional tool to probe the double-layer structure inside of porous electrodes and application in the modeling of capacitive deionization. J. Phys. Chem. Lett. 1, 205–210 (2009).

[b62] Andelman M. D., Walker G. S., inventors; Biosource, Inc., assignee. Charge barrier flow-through capacitor. United States patent US 6,709,560. March 23 2004.

[b63] Lee J.-B., Park K.-K., Eum H.-M. & Lee C.-W. Desalination of a thermal power plant wastewater by membrane capacitive deionization. Desalination 196, 125–134 (2006).

[b64] Li H. B. *et al.* Electrosorptive desalination by carbon nanotubes and nanofibres electrodes and ion-exchange membranes. Water Res. 42, 4923–4928 (2008). 18929385 10.1016/j.watres.2008.09.026

[b65] Biesheuvel P. & Van der Wal A. Membrane capacitive deionization. J. Membr. Sci. 346, 256–262 (2010).

[b66] Gao Y. *et al.* Electrosorption behavior of cations with carbon nanotubes and carbon nanofibres composite film electrodes. Thin Solid Films 517, 1616–1619 (2009).

[b67] Li H. *et al.* A comparative study on electrosorptive behavior of carbon nanotubes and graphene for capacitive deionization. J. Electroanal. Chem. 653, 40–44 (2011).

