# Supplementary material for: Facile synthesis of novel graphene sponge for high performance capacitive deionization
Source: Sci Rep. 2015 Feb 13;5:8458. doi: 10.1038/srep08458 (PMC4327409; doi:10.1038/srep08458)
Supplement: Supplementary Information — includes Figure and Tables [file srep08458-s1.doc]

**Facile synthesis of novel graphene sponge for high performance capacitive deionization**

Xingtao Xu ^a^, Likun Pan a[[1]](#footnote-2)*, Yong Liu ^a^, Ting Lu ^a^, Zhuo Sun ^a^ and Daniel H. C. Chua^b^

^a^Engineering Research Center for Nanophotonics & Advanced Instrument, Ministry of Education, Department of Physics, East China Normal University, Shanghai 200062, China

^b^Department of Materials Science and Engineering, National University of Singapore, Singapore 117574

**Supplementary Figures**


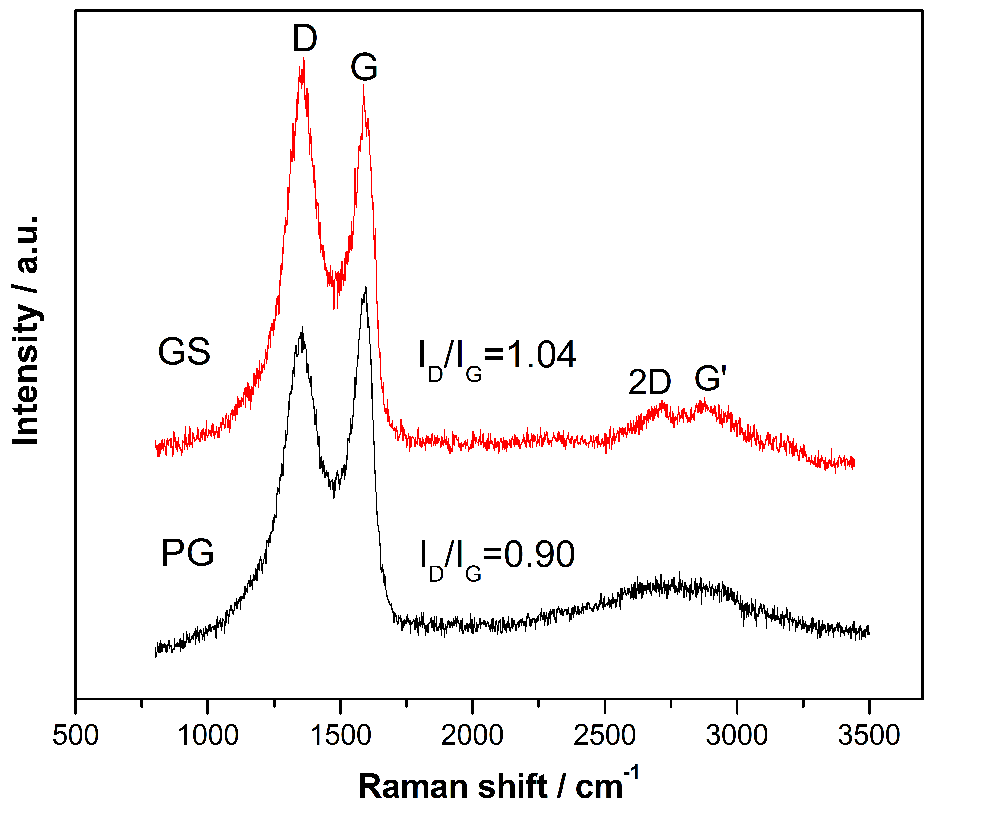


**Supplementary Figure 1** Raman spectra of GS and PG.

**Supplementary Tables**

**Supplementary Table 1** Comparison of electrosorption capacities of various graphene electrodes.

| Sample | Applied voltage (V) | | Initial NaCl conductivity (µS cm^-1^) | | Electrosorption capacity (mg g^-1^) | | Specific surface area (m^2^ g^-1^) | |
| --- | --- | --- | --- | --- | --- | --- | --- | --- |
| Graphene[^1^](#_ENREF_1) | 2.0 | | ~50 | | 1.85 | | 14.2 | |
| Pyridine–thermal prepared graphene[^2^](#_ENREF_2) | 2.0 | | ~87 | | 0.88 | | - | |
| Graphene-like nanoflakes[^3^](#_ENREF_3) | 2.0 | | ~57 | | 1.36 | | 222 | |
| 3D-macroporous graphene architecture[^4^](#_ENREF_4) | 1.6 | | ~105 | | 3.9 | | 339 | |
| Sponge-templated graphene[^5^](#_ENREF_5) | | 1.5 | ~106 | 4.95 | | 305 | |  |
| Pristine graphene (this work) | 1.5 | | ~100 | | 2.36 | | 150.5 | |
| Graphene sponge (this work) | 1.5 | | ~100 | | 5.52 | | 356 | |

**Supplementary Table 2** Coefficients of kinetic equations for the electrosorption of NaCl by GS and PG.

| Sample |  | GS | PG |
| --- | --- | --- | --- |
| Pseudo-first-order  kinetic equation | k_1_ | 0.180 | 0.128 |
|  | r^2^ | 0.978 | 0.931 |
| Pseudo-second-order  kinetic equation | k_2_ | 0.036 | 0.028 |
|  | r^2^ | 0.989 | 0.931 |

**Supplementary Table 3** Comparison of electrosorption capacities of various carbon electrodes.

| Sample | Applied voltage (V) | Initial NaCl concentration (mg L^-1^) | Electrosorption capacity (mg g^-1^) | Specific surface area (m^2^ g^-1^) |
| --- | --- | --- | --- | --- |
| AC[^6^](#_ENREF_6) | 1.2 | ~500 | 9.72 | 1153 |
| Multi-walled CNTs[^7^](#_ENREF_7) | 1.2 | ~3000 | 1.7 | 129.4 |
| MC[^8^](#_ENREF_8) | 1.2 | ~4460 | 14.5 | 488 |
| Microporous CA monoliths[^9^](#_ENREF_9) | 1.25 | ~2900 | 9.6 | ~500 |
| CA[^10^](#_ENREF_10) | 1.3 | ~2000 | 7.1 | 113 |
| CNTs/graphene composite[^11^](#_ENREF_11) | 1.2 | ~500 | 1.4 | 438.6 |
| AC/graphene composite[^12^](#_ENREF_12) | 1.2 | ~500 | 2.94 | 779 |
| CNTs[^13^](#_ENREF_13) | 1.2 | ~3500 | 9.35 | 153 |
| Graphene aerogel[^14^](#_ENREF_14) | 1.2 | ~500 | 9.9 | - |
| GS (this work) | 1.2 | ~500 | 14.9 | 356.0 |
| PG (this work) | 1.2 | ~500 | 4.64 | 150.5 |

**Supplementary Table 4** Parameters determined from electrosorption isotherms of GS and PG.

| Electrode | Langmuir parameters | | |  | Freundlich parameters | | |
| --- | --- | --- | --- | --- | --- | --- | --- |
|  | q_m_ | K_L_ | r^2^ |  | n | K_F_ | r^2^ |
| GS | 24.5 | 0.0028 | 0.995 |  | 2.67 | 1.28 | 0.922 |
| PG | 7.9 | 0.0029 | 0.980 |  | 2.69 | 0.42 | 0.956 |

**References**

1 Li, H. B., Lu, T., Pan, L. K., Zhang, Y. P. & Sun, Z. Electrosorption behavior of graphene in NaCl solutions. *J. Mater. Chem.* **19**, 6773-6779, (2009).

2 Wang, H. *et al.* Graphene prepared via a novel pyridine–thermal strategy for capacitive deionization. *J. Mater. Chem.* **22**, 23745-23748 (2012).

3 Li, H., Zou, L., Pan, L. & Sun, Z. Novel graphene-like electrodes for capacitive deionization. *Environ. Sci. Technol.* **44**, 8692-8697 (2010).

4 Wang, H. *et al.* Three-dimensional macroporous graphene architectures as high performance electrodes for capacitive deionization. *J. Mater. Chem. A* **1**, 11778-11789 (2013).

5 Yang, Z. Y. *et al.* Sponge‐templated preparation of high surface area graphene with ultrahigh capacitive deionization performance. *Adv. Funct. Mater.* **24**, 3917-3925 (2014).

6 Chen, Z., Song, C., Sun, X., Guo, H. & Zhu, G. Kinetic and isotherm studies on the electrosorption of NaCl from aqueous solutions by activated carbon electrodes. *Desalination* **267**, 239-243 (2011).

7 Dai, K., Shi, L., Fang, J., Zhang, D. & Yu, B. NaCl adsorption in multi-walled carbon nanotubes. *Mater. Lett.* **59**, 1989-1992 (2005).

8 Tsouris, C. *et al.* Mesoporous carbon for capacitive deionization of saline water. *Environ. Sci. Technol.* **45**, 10243-10249 (2011).

9 Suss, M. E. *et al.* Capacitive desalination with flow-through electrodes. *Energy Environ. Sci.* **5**, 9511-9519 (2012).

10 Xu, P., Drewes, J. E., Heil, D. & Wang, G. Treatment of brackish produced water using carbon aerogel-based capacitive deionization technology. *Water Res.* **42**, 2605-2617 (2008).

11 Li, H., Liang, S., Li, J. & He, L. The capacitive deionization behaviour of a carbon nanotube and reduced graphene oxide composite. *J. Mater. Chem. A* **1**, 6335-6341 (2013).

12 Li, H. B., Pan, L. K., Nie, C. Y., Liu, Y. & Sun, Z. Reduced graphene oxide and activated carbon composites for capacitive deionization. *J. Mater. Chem.* **22**, 15556-15561, (2012).

13 Wang, S. *et al.* Equilibrium and kinetic studies on the removal of NaCl from aqueous solutions by electrosorption on carbon nanotube electrodes. *Sep. Purif. Technol.* **58**, 12-16 (2007).

14 Yin, H. *et al.* Three‐dimensional graphene/metal oxide nanoparticle hybrids for high‐performance capacitive deionization of saline water. *Adv. Mater.* **25**, 6270-6276 (2013).

1. * Corresponding author. Tel: 86 21 62234132; Fax: 86 21 62234321; E-mail: lkpan@phy.ecnu.edu.cn [↑](#footnote-ref-2)
